# Antimicrobial Resistance Patterns, Extended-Spectrum Beta-Lactamase Production, and Associated Risk Factors of *Klebsiella* Species among UTI-Suspected Patients at Bahir Dar City, Northwest Ethiopia

**DOI:** 10.1155/2022/8216545

**Published:** 2022-03-18

**Authors:** Asmamaw Ameshe, Tigist Engda, Mucheye Gizachew

**Affiliations:** ^**1**^ Medical Microbiology Department, Kobo District Hospital, Amhara Regional State, Wollo, Ethiopia; ^2^Department of Medical Microbiology, School of Biomedical and Laboratory Sciences, College of Medicine and Health Sciences, University of Gondar, Gondar, Ethiopia

## Abstract

**Introduction:**

*Klebsiella* species cause pneumonia, UTI, and septicemia in human beings. Beta-lactam drugs are used extensively to treat patients infected with *Klebsiella*, but most of the *Klebsiella* species are resistant to third- and fourth-generation cephalosporins and monobactams to which data are scarce in the study area.

**Objective:**

To determine the prevalence, antimicrobial resistance, ESBL production, and associated risk factors of *Klebsiella* species among UTI-suspected patients in Bahir Dar City, Northwest Ethiopia.

**Methods:**

A multi-institution-based prospective cross-sectional study was conducted from January to May 2019. Midstream urines were collected from 385 patients and inoculated onto CLED and MacConkey agars. Identification of growth was done by a battery of biochemical tests. Antimicrobial resistance and ESBL production patterns were determined by using the disc-diffusion method on Mueller–Hinton agar. Quality of data was maintained by following SOPs and using *Klebsiella pneumoniae* (ACTT700603). Logistic regression statistical analysis was done using the SPSS, version 25, statistical package. A *pvalue ≤ 0.05* was considered statistically significant.

**Results:**

The median age of the study participants was 32 years. Majority of them were female, urban residents, and unable to read and/or write. The total *Klebsiella* species detected were 38 (9.9%). Of which, 25 (65.8%) were *Klebsiella pneumoniae*, followed by 6 (15.8%) *Klebsiella ozaenae*. 20 (80%), 19 (76%), and 19 (76%) *Klebsiella pneumoniae* showed resistance to amoxicillin/clavulanic acid, ampicillin, and cotrimoxazole, respectively. All *K. oxytoca* were resistant to ampicillin, and all *K. rhinoscleromatis* were resistant to cefoxitin and cefotaxime. *Klebsiella species* that showed resistance to ≥3 antimicrobials were 26 (68%). ESBL-producing *Klebsiella* species were 10 (26.3%). Patients who had history of antibiotic use, hospitalization, and tight dressing habit had more risk of getting UTI (*p* < 0.05) than their counterparts.

**Conclusions:**

Overall UTI prevalence in our study was lower than that of previous Ethiopian studies. High MDR and ESBL-producing *Klebsiella* species were detected. Patients' history of antibiotic use, hospitalization, and tight dressing habit were risk factors for UTI. It calls up for improving prevention/control systems of *Klebsiella* species.

## 1. Introduction

Urinary tract infection (UTI) caused by bacteria is one of the major reasons for visiting health institutions, morbidity, and comorbidities in patients with underlying conditions worldwide [1–3]. Though different pathogens cause UTI, various studies described that the causative agents of UTI are predominantly associated with Gram-negative bacteria such as *E. coli*, *Pseudomonas* species, *Klebsiella* spp., *Proteus* spp., and Gram-positive bacteria such as coagulase-negative staph (CoNS) and *S. aureus* [2, 4–6].

The prevalence of *Klebsiella* species isolated from urine specimens in Nepal revealed that *K. pneumoniae* accounted for 145 (2.3%) [5]; whereas the prevalence of *Klebsiella* species in India was 170 (4.3%), of which, 116 (68.2%) were *K. pneumoniae* and 54 (31.8%) were *K. oxytoca* [3]; in Saudi Arabia 23.5% [7]; Yemen 42 (24.7%) [8]; and Nigeria 14 (7.0%) were *Klebsiella pneumoniae* and 2 (1.0%) were *Klebsiella oxytoca* [3]. In Uganda, 10/86 (11.6%) *Klebsiella pneumoniae* and 6/86 (7.0%) Klebsiella oxytoca were isolated from midstream urine of the patients [2]; in the northeastern part of Ethiopia, Klebsiella species isolated were 40 (5.88%) among the urinary isolates [4]; 8.1% Klebsiella spp. in Northern Ethiopia (Mekele) [1]; 29 (15.8%) K. pneumoniae in Gondar [9]; and two studies in Bahir Dar showed 13.6% K. pneumoniae [10] and 5/7 (71.4%) K. pneumoniae, 1/7 (14.3%) K. ozaenae, and 1/3 K. rhinoscleromatis [11].

The World Health Organization (WHO) has declared that multidrug resistance (MDR) is one of the top 10 global public health threats faced by humanity and the problem induces about 700,000 deaths every year in the world [12]. It is found that patients with infections caused by drug-resistant bacteria are at increased hospital stay and risk of worse clinical outcomes, which may extend up to death and costs more than patients infected with nonresistant strains of the same bacteria [13, 14]. In addition, antimicrobial resistance increases resource need at health institutes specifically and at the society level largely [15]. The influence and the problem are high in low-income countries including Ethiopia due to the high prevalence of infection, irrational uses of antimicrobials, over-the-counter availability of different antimicrobials, and absence of well-equipped laboratory facilities for antimicrobial susceptibility testing [13, 16, 17].

Thus, knowing of the current antimicrobial-resistant patterns of *Klebsiella* spp. has a paramount importance for the better management of UTI patients. Literature indicated that *K. pneumoniae* are 100% resistant to ceftriaxone, ceftazidime, ciproﬂoxacin, and Augmentin and 71.4% resistant to cefotaxime [18]. Similarly, a Nigerian study revealed that all *Klebsiella* spp. isolated from urine specimen were resistant to ampicillin [16]. A report in Ethiopia where *Klebsiella* species isolated from UTI-suspected patients demonstrated 66.7% resistance to cotrimoxazole and 100% to ampicillin. But isolates exhibited a lower resistance rate (5.6% to 22.2%) to cefoxitin, amoxicillin-clavulanate, ciprofloxacin, and gentamicin [19]. In addition, increasing ESBL-producing microorganisms in recent years have resulted in limitations of treatment options [20].

Understanding of risk factors associated with UTIs in human beings can facilitate physicians tailor appropriate prophylactic approaches [21]. Studies revealed that there was a high incidence of UTIs among sexually active young women, which was associated with recent sexual intercourse and use of a diaphragm with spermicide, as well as with a history of recurrent UTIs. Lack of postcoital urination, vaginal douches, use of hot tubs, restrictive underwear, and the hygiene and circumcision status of male partners have been proposed as risk factors [22, 23]. So, this study was intended to determine the prevalence, antibiogram, ESBL production patterns, and its associated risk factors of *Klebsiella* species isolated from urinary tract infection-suspected patients.

## 2. Materials and Methods

### 2.1. Study Design, Period, and Area

A multicenter institution-based cross-sectional study was conducted from January to March 2019 at Felege-Hiwot Comprehensive Specialized Hospital, Gammby Private General Hospital, and Bahir Dar Health Center, Bahir Dar City, Amhara National Regional State. Bahir Dar City, the capital of the Amhara National Regional State, is located 576 km northwest from the capital city of the country, Addis Ababa. Two comprehensive specialized referral hospitals, eight health centers, three private hospitals, and seven private specialized clinics provide health services to the community. Among these, one comprehensive specialized hospital, one health center, and one general private hospital were selected for this study. Felege-Hiwot Referral Hospital is a tertiary care referral hospital serving over 7 million people from the surroundings with 500 beds, 9 operating tables, and over 600 employers. Gammby General Hospital has 150 beds and 2 operating tables and provides service for around 2 million people. Both hospitals provide obstetrics, pediatrics, internal medicine, ophthalmology, orthopedics, and surgery services, whereas the Bahir Dar Health Center provides service for outpatients of acute cases and delivery services for around 3 million people.

### 2.2. Sampling Technique of Institutions and Sample Size Determination

A simple random sampling method was employed to select two hospitals and one health center. UTI patients who attended one month before the study period the selected health institutions were found to be 395, and the estimated number of patients during the data collection period, from January to March 2019, was 1185. Therefore, 384 study participants were allocated to the three health institutions proportionally based on their patient flow in such a way that 277 were from Felege-Hiwot Comprehensive Specialized Hospital, 83 from Gammby Private General Hospital, and 24 from Bahir Dar Health Center.

### 2.3. Source Population

All patients who were attending their medical treatments at the three selected health institutions at Bahir Dar City during the study period were the source population.

### 2.4. Study Population

Suspected patients for UTI and who were attending their medical treatments at the three selected health institutions at Bahir Dar City during the study period were the study population.

### 2.5. Inclusion Criteria

All UTI-suspected patients who attended the selected health institutions during the study period and who did not take any antibiotics with in the previous two weeks and were voluntary to participate on the study were included in the study.

### 2.6. Sociodemographic Data and Specimen Collection

After informed consent/assent was obtained, a predesigned structured questionnaire was administered to collect the sociodemographic variables (age, sex, marital status, education level, occupation, residence) and clinical risk factors (history of catheterization, history of UTI, history of previous hospitalization, and history of antibiotic use). To ensure the reliability of information, all the questionnaires were administered by native Amharic speakers and were checked for completeness and consistency every day. The patients or patients' guardians were given a sterilized container to collect midstream urine and were transported to microbiology laboratory for examination.

### 2.7. Culture and Identification

Each urine sample was inoculated onto cystine-lactose-electrolyte-deficient (CLED) agar and incubated at 37°C for 24 hours. The counted colonies yielding bacterial growth of 10^5^ CFU/mL for single midstream urine was considered significant for positive bacteriuria. Pure colony was transferred to MacConkey and incubated at 37°C for further 24 hours. Isolates were preliminarily screened by their colony morphology, pigment production (pink), flat or mucoid colonies, and Gram staining reaction. Further identifications of isolates were made by doing a battery of biochemical tests such as triple sugar iron agar, indole, Simon's citrate, and urea, and motility tests were performed by picking lactose fermenter or pink colonies from MacConkey agar for identification of isolates to the species level by using the Clinical Laboratory Standards Institute (CLSI) guideline 2018 [24].

### 2.8. Antimicrobial Resistance Testing

The antimicrobial resistance testing was done by using the Kirby-Bauer disc-diffusion method on Mueller–Hinton agar by following the CLSI guideline 2018 [24] for ampicillin (10 *µ*g), amoxicillin/clavulanic acid (20 *µ*g), ciprofloxacin (5 *µ*g), gentamicin (10 *µ*g), cotrimoxazole (25 *µ*g), chloramphenicol (30 *µ*g), nitrofurantoin (300 *µ*g), meropenem (10 *µ*g), ceftazidime (30 *µ*g), cefotaxime (30 *µ*g), and cefoxitin (30 µg) discs (Oxoid, UK). A suspension of pure colony from each confirmed growth was inoculated into sterile normal saline and incubated at 37°C for 15 to 30 minutes. The suspension was adjusted to 0.5% McFarland standard. A sterile cotton applicator stick was used to uniformly distribute the suspension on Mueller–Hinton agar (Oxoid Ltd., UK). After incubation at 37°C for 24 hours, the zones of inhibition were measured using a caliper. Results were measured, recorded, and classified as susceptible, intermediate, and resistant by using the CLSI 2018 performance standard for antimicrobial susceptibility testing interpretation [24]. Here, the MDR isolates were defined as isolates which acquired nonsusceptibility to at least one agent in ≥3 classes of antimicrobials [25].

### 2.9. Extended-Spectrum *β*-Lactamase Detection

The screening for extended-spectrum beta-lactamase (ESBL) was done by using ceftazidime (≤22 mm) and cefotaxime (≤27 mm). More than one antimicrobial discs were used for screening to improve the sensitivity of ESBL detection as recommended by CLSI guideline 2018 [24].

The organisms that showed zone of inhibition lower than the minimum for any of the above antimicrobial discs were considered as ESBL positive. The phenotypic confirmations were done by testing the isolated bacterial species against ceftazidime (30 *μ*g) and ceftazidime-clavulanic acid combination (30/10 *μ*g/discs) (Oxoid Ltd., UK). In this test, an overnight culture suspension of the bacterial isolate was adjusted to 0.5 McFarland's standard. Lawn cultures were done on the Mueller–Hinton agar (MHA) plate. The ceftazidime and ceftazidime-clavulanic acid discs were placed at 20 mm apart on the agar surface. After incubating at 37°C for 24 hours, a ≥5 mm increase in the zone diameter in comparison to ceftazidime were taken as indicative for ESBL positive/producer.

### 2.10. Quality Control

The questionnaire was prepared in English and translated into the local language which the study participants use (Amharic), and then it was translated back to English for analysis and report. It was pretested before the commencement of the actual study to make sure that whether the questionnaire was appropriate and understandable to the study participants. Microbiology standard operating procedures were strictly followed throughout the laboratory processes. *Escherichia coli* ATCC25922 as an ESBL-negative and *Klebsiella pneumoniae* ATCC700603 as ESBL-positive reference strains were used.

### 2.11. Data Analysis

Data were entered and coded in EpiData and exported and analyzed using SPSS software (version 25). The characteristics of the study patients were summarized using frequencies and mean and standard deviation. Binary logistic regression was done to determine the association of independent and dependent variables. Adjusted odds ratios were computed using multivariable logistic regression for variables with *p* value < 0.2 in the crude odds ratio. Findings of the study were presented in tables and figures. A *pvalue* ≤ 0.05 was considered statistically significant.

### 2.12. Ethical Consideration

This study was ethically approved by the Ethical Review Committee of the School of Biomedical and Laboratory Sciences, University of Gondar. Moreover, legal permission was obtained from the Felege-Hiwot Comprehensive Specialized Hospital, Gammby Private General Hospital, and Bahir Dar Health Center of higher management prior to data collection. Written consent and assent were taken from each participant after they understood the purpose of the study. Data were kept in full confidentiality and were not disclosed to an unauthorized person. Positive cases were referred to the attending physician for their better management.

## 3. Results

### 3.1. Sociodemographic Characteristics

A total of 385 clinically UTI-suspected patients were included in the study; of these, 205 (53.2%) were females, and majority of the study participants (37.9%) were in the age groups of 16–30 years with median age of 32 years. Moreover, 60.8% of the participants were from urban areas and 24.4% were illiterate (Table 1).

### 3.2. Prevalence of *Klebsiella* Species

**Figure 1:**
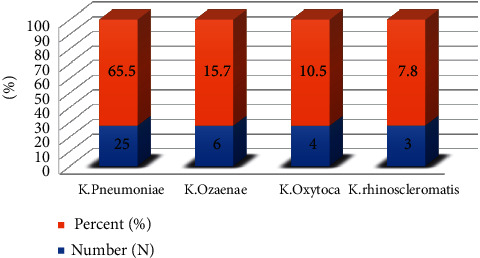
Distribution of *Klebsiella* species isolated from UTI-suspected patients who attended Bahir Dar City health institutions, Bahir Dar, Northwest Ethiopia, 2019.

The prevalence of *Klebsiella* species isolated from the 385 study participants examined in this study was 38 (9.9%). Of the positive culture, 25 (65.8%) were *Klebsiella pneumoniae*, 4 (10.5%) *K. oxytoca*, 6 (15.8%) *K. ozaenae*, and 3 (7.9%) *K. rhinoscleromatis*. Majority (22, 57.9%) of the total isolates were from female study participants. The distribution frequency of *Klebsiella* species in each health institutions was 25 from Felege-Hiwot Comprehensive Specialized Hospital, eight from Gammby Private General Hospital, and five from Bahir Dar Health Center (Figure 1).

### 3.3. Antimicrobial Resistance Patterns of *Klebsiella* Species

As illustrated in Table 2, 20 (80%), 19 (76%), 19 (76%), 8 (32%), 7 (28%), 7 (28%), and 6 (24%) *Klebsiella pneumoniae* isolates were resistant to amoxicillin/clavulanic acid, ampicillin, cotrimoxazole, gentamicin, nitrofurantoin, cefoxitin, and cefotaxime, respectively; while 22 (88%), 18 (72%), 17 (68%), and 16 (64%) of the isolates were sensitive to meropenem, chloramphenicol, ceftazidime, and ciprofloxacin, respectively. Six (100%), 5 (83.3%), and 5 (83.3%) *K. ozaenae* isolates were resistant to ampicillin, amoxicillin/clavulanic acid, and cotrimoxazole, respectively; however, 5 (83.3%) were sensitive to cefotaxime, gentamicin, chloramphenicol, and nitrofurantoin. On the other hand, all or 4 (100%) *K. oxytoca* isolates were resistant to ampicillin but sensitive to meropenem and cefotaxime. Moreover; all the *K. rhinoscleromatis* (3 (100%)) isolates were sensitive to cefoxitin and cefotaxime.

### 3.4. Multidrug Resistance Patterns of the Isolates

Twenty-six (68%) of the *Klebsiella* species isolated were resistant to three or more antimicrobial drugs, and thus, they exhibited MDR. Of which, 14 (36.8%), 6 (15.8%), 3 (7.9%), 2 (5.3%), and 1 (2.6%) showed resistance to three, four, five, nine, and seven antimicrobials, respectively. Regarding *Klebsiella pneumoniae*, 15 (39.5%) of the isolates showed MDR to three to nine antimicrobials, and of which, 5 (13.2%), 4 (10.5%), 3 (7.9%), 2 (5.3%), and 1 (2.6%) showed resistance to three, four, five, nine, and seven antimicrobial drugs, respectively (Table 3).

### 3.5. Extended-Spectrum Beta-Lactamase Production Patterns of Isolated *Klebsiella* Species

Among the total *Klebsiella species* identified, 10 (26.3%) were ESBL producer. Based on the 2018 CLSI guideline, 38 isolates were checked for the confirmation of ESBL production by the combined disc-diffusion method. All the ESBL-producing species were found to be *Klebsiella pneumoniae*, in which 5 (50%), 3 (30%), and 2 (20%) isolates were found in the age group of 16–30, 46–60, and 6–15 years, respectively. Moreover, 6 (60%) were from the female participants. Extended-spectrum beta-lactamase-producing *Klebsiella pneumoniae* species showed resistance to ampicillin (100%), amoxicillin/clavulanic (100%), TMP-SMX (100%), ciprofloxacin (50%), gentamicin (40%), nitrofurantoin (50%), cefoxitin (40%), cefotaxime (50%), ceftazidime (50%), and both meropenem and chloramphenicol (2/10, 20%). Of the ESBL-producing *Klebsiella pneumoniae* isolates, one isolate was sensitive to cefotaxime, three were intermediate, and the remaining seven were resistant to cefotaxime (Table 4).

### 3.6. Risk Factors Associated with Urinary Tract Infections

A multivariable logistic regression analysis was done to evaluate the associated risk factors for UTI. Such a statistical analysis showed that pervious antibiotic use, history of hospitalization, and habit of tight dressing were statistically significant for getting UTI. The result showed that those study participants who had pervious antibiotic use were 2.618 times (AOR = 2.618, 95% CI: 1.18–6.129) more likely to have UTI compared with their counterparts. Similarly, patients who had history of previous hospitalization had 5.873 times (AOR = 5.873, 95%CI: 2.355–14.6) more likely to have a risk for UTI than those participants who did not have hospitalization history. Moreover, the study participants who had the habit of wearing tight dresses were 15.038 times (AOR = 15.038, 95%CI: 1.7–39) more likely to acquire UTI than their counterparts (Table 5).

## 4. Discussion

This multi-institution study was conducted to determine prevalence, antimicrobial resistance, ESBL production patterns, and associated risk factors of *Klebsiella* species among UTI-suspected patients. In the present study, the overall prevalence of *Klebsiella* species was 38 (9.9%), which is in line with Ethiopian (8.1% to 10.9%) [1, 19, 26] and Nigerian (7.3%) [27] and (8%) [6] reports. But the result of the present study is lower than that of previous reports in Ethiopia (11/74, 14.9%) [28] and (27.9%) [29], Uganda (18.6%) [2], Iraq (15%) [30], and India (14.72%) [31]. The prevalence of *Klebsiella* species isolated in the current study is higher than that of other studies conducted in Nepal (2.29%) [5] and (4.4%) [20] and India (4.3%) [3].

Of all the isolates of *Klebsiella* species in the present study, 25 (65.8%) were *Klebsiella pneumoniae*, followed by *K. oxytoca* 6 (15.8%), which is higher than previous reports in Saudi Arabia (21.5%) [32] and (23.5%) [7], Yemen 42 (24.7%) [8], Nigeria (7%) [6], Uganda (11.6%) [25], and Ethiopia: Addis Ababa (7%) [19], Dessie (5.88%) [4], and Bahir Dar (13.6%) [10]; whereas in Gondar, 29 (15.8%) *K. pneumoniae* were isolated [9]; however, our results are in agreement with those of other reports from Ethiopia where 5/7 (71.4%) were *K. pneumoniae*, 1/7 (14.3%) were *K. ozaenae*, and *K. rhinoscleromatis* each [11]; and in Iraq where 28 (85%) of the isolates have been identified as *K. pneumoniae* and only 5 (15%) isolates were detected as *K. oxytoca* [30]. Out of the 16 *Klebsiella* species isolated, 14 (7.0%) were biochemically identified as *Klebsiella pneumoniae* and 2 (1.0%) were *Klebsiella oxytoca* in Nigeria [6]. *K. ozaenae* (4, 10.5%) and *K. rhinoscleromatis* (3, 7.9%) were also identified in the current study, which were not detected in the Iraq and Nigerian studies stated above. The present study showed that majority (22, 57.9%) of the total isolates causing UTI were from female participants, which is in line with studies that demonstrated 81.2% of culture positivity of urine specimen were from female patients in Ethiopia [19]; 29 (88%) of *Klebsiella* species isolated were from the female participants in Iraq [30], and 70 (60.35%) of UTI were recorded in females in Saudi Arabia [32]. However, a report from Yemen indicated that the majority (55.9%) of culture positivity of UTI was obtained from the male study participants [8]. These discrepancies in the rates of isolation of *Klebsiella* species causing UTI in different studies might be resulted from variations in the sample size and type of the populations studied, laboratory methods practiced, subjectivity on definition of significant bacteriuria during culture reading, the length of the study period, and specimen's quality. In addition, the possible reason of gender variability of UTI prevalence might be related with the history of urinary tract operation, no use of antibiotics in the preceding time periods and infection outside the urinary tract [33]; and the other cause might be due to the difference in the target setting/population [34], where those male patients might have different predisposing factors such as age (prevalence may range from 20% to 50% in old age), enlarged prostate, prostatism, debilitation, and subsequent instrumentation of the urinary tract. On the other hand, the high prevalence of UTI in females is unambiguously due to anatomical factors such as the shorter urethra and the closeness of the urethral opening to the anus that shortens the distance that bacteria must travel to reach the bladder and sexual intercourse, bad personal hygiene, and incontinence.

*Klebsiella* species identified in the current study showed high levels (66.7% to 100%) of resistance to ampicillin, amoxicillin-clavulanate, and cotrimoxazole. On the other hand, meropenem, chloramphenicol, ciprofloxacin, ceftazidime, cefoxitin, cefotaxime, gentamicin, and nitrofurantoin were found to be the drug of choice for *Klebsiella* species associated UTI therapy since the isolates showed better sensitivity (0% to 28% resistance) to these antimicrobials tested. A similar result was reported in Ethiopia where *Klebsiella* species isolated from UTI-suspected patients demonstrated 66.7% resistance to cotrimoxazole and 100% to ampicillin. However, isolates demonstrated a lower resistance rate (5.6% to 22.2%) to cefoxitin, amoxicillin-clavulanate, ciprofloxacin, and gentamicin [19]. Isolates in the present study showed better sensitivity to nitrofurantoin but higher resistance to amoxicillin-clavulanate, which is different from Bitew et al report where isolates showed better sensitivity to amoxicillin-clavulanate but higher resistance to nitrofurantoin [19]. These variations might be because of the differences in the levels of consciousness of the inhabitants on how to use antimicrobials, and unavailability of bacteriology laboratory facilities, habit of irrational use of antimicrobials (use of broad-spectrum antimicrobials empirically and from over the counter), and unavailability of nitrofurantoin were more encountered in the setting of the present study (730 km far from Addis Ababa) compared with the capital of the country where Bitew et al.'s work was done. In line with the current results, the studies in the northwestern part of Ethiopia revealed that *K. pneumoniae* showed a high level of susceptibility to meropenem 5/5 (100%), and ceftazidime and nitrofurantoin 4/5 (80%) each [11]; and in the southern part of Ethiopia, 91% to 100% of the *Klebsiella* species isolated were susceptible to gentamicin, amikacin, nitrofurantoin, doxycycline, and ceftriaxone [35]; and a study in Yemen also revealed that isolates causing UTI were resistant to ampicillin (95.2%) and amoxicillin (97.6%) but less resistant to imipenem (21.7%), nitrofurantoin (26.5%), amikacin (28.9%), and ertapenem (30.1%) [8]. Another study showed that maximum active drugs against *Klebsiella* species (*K. pneumoniae*) are amikacin, nitrofurantoin, and meropenem, showing 57.1% activity. *K. pneumoniae* strains are 100% resistant to ceftriaxone, ceftazidime, ciproﬂoxacin, and Augmentin and 71.4% resistant to cefotaxime [18]. Besides chloramphenicol, ceftazidime, and ciprofloxacin, results of the present study indicate that meropenem is one of the most effective antimicrobials against *Klebsiella* species since the isolates demonstrated susceptibility ranging from 66.7% to 100%. This was in agreement with another study in India where 65.7% of *K. pneumoniae* were meropenem susceptible [36]. A 2018 study conducted in Germany also revealed that the carbapenem nonsusceptibility in *K. pneumoniae* isolates is still low even though it is slowly growing and in the light of the strong increase of *K. pneumoniae* isolates over the last year; this poses a significant challenge to public health [37].

Twenty-six (68%) of the *Klebsiella* species isolated in the current study were resistant to ≥3 antimicrobial drugs, and thus, they exhibited MDR. In addition, 15 (39.5%) of *Klebsiella pneumoniae* exhibited MDR (resistant to ≥3 antimicrobials), which is nearly in line with a study done at Dessie, Ethiopia, where 63% of *Klebsiella* spices showed MDR [26]; however, it is lower than another study conducted in Northwest Ethiopia where the highest MDR was observed in *Klebsiella* specie 6/7 (85.7%) [11]; and in Equatorial Guinea, 91.7%, 33.3%, and one isolate of *K. pneumoniae* demonstrated MDR, extensive drug resistance (XDR), and pan-drug resistance (PDR), respectively [38]. Rates of MDR *K. pneumoniae* in the current study are higher than a report from Iran where 96/198 (48.5%) of the isolates exhibited MDR and 14/96 (14.6%) of these MDR isolates were completely (100%) resistant to all antibiotics investigated [39].

In the case of ESBL production patterns, results of the present study indicates 10/25 (40%) prevalence of ESBL-producing *K. pneumoniae*, which is slightly in agreement with a study conducted in India where 44.7% *Klebsiella pneumoniae* were ESBL producers by using MIC [40] but is different from previous findings in Ethiopia where (1/7, 14.3%) [11], (4, 23.5%) [28], and 76.4% of *K. pneumoniae* were ESBL producers [41]; in Sri Lanka, *Klebsiella pneumoniae* with 4 (25%) ESBL producers [42]; Bangladesh, 18.57% *Klebsiella* spp. ESBL [43]; Pakistan, 15.25% (9/59) in *K. pneumoniae* [44]; and Iraq 85.7% (66) of *K. pneumoniae* isolated from UTI patients were ESBL producers based on phenotyping ESBL detection by ESBL E-strip tests [45]. One of the possible reasons for the high rates of resistance to routinely prescribed antimicrobials observed in the current study might be associated with the occurrence of substantial numbers of ESBL producer isolates. An irrational and frequent use of these antimicrobials, easy availability, and the over-the-counter sale of the antimicrobials without a right prescription and inappropriate dosing schedule might also attribute to the occurrence of these high rates of resistance.

Though statistically not associated, being women, old age, rural residency, lower educational status, farmer, and inpatient were with high prevalence of UTI compared with their counterparts. However, history of antibiotic use, previous hospitalization, and tight dressing habit were independent associated risk factors of UTI in the current study (*p* < 0.05). It is supported by a previous study reported in India where previous antibiotic exposure was identified as a risk factor of getting UTI and ESBL-producing organisms [46]. Similarly, a study in Israel showed that the length of any hospital stay is significantly associated with UTIs [47].

## 5. Conclusion and Recommendation

The prevalence of *Klebsiella* spp. in this study is comparatively higher than that of many other studies reported across the globe. *Klebsiella pneumoniae, K. ozonae, K. rhinoscleromatis,* and *K. oxytoca* were identified and of these, K. pneumoniae was the predominant isolate. More than two-thirds of isolates showed MDR. The rate of ESBL-producing K. *pneumoniae* is higher than earlier Ethiopian reports. Chloramphenicol, ceftazidime, ciprofloxacin, and meropenem remain the most effective antimicrobials for UTI. History of antibiotic use, earlier hospitalization, and tight dressing habit were risk factors associated with UTI (*p* < 0.05). Thus, at least minimizing empirical treatment of UTI by using urine culture and awaiting antimicrobial susceptibility reports are compulsory.

## Figures and Tables

**Table 1:** Sociodemographic characteristics of UTI-suspected patients who attended Bahir Dar City health institutions, Bahir Dar, Northwest Ethiopia, 2019.

Variables	Categories	Number of participants (N)	Percentage
Gender	Male	180	46.8
	Female	205	53.2

Age group, years	<5	20	5.2
	6–15	44	11.4
	16–30	146	37.9
	31–45	96	24.9
	46–60	55	14.3
	>60	24	6.2

Level of education (educational status)	Illiterate	94	24.4
	Primary school	86	23.3
	Secondary school	107	27.8
	College and university	98	25.5

Health facility	Felege-Hiwot	278	72.2
	Gammby	87	22.6
	Bahir Dar Health Center	20	5.2

Residency	Urban	234	60.8
	Rural	151	39.2

Occupational status	Merchant	29	7.5
	Housewife	112	29.1
	Civil	102	26.5
	Farmer	71	18.4
	Others	71	18.4

**Table 2:** Antimicrobial resistance patterns of *Klebsiella* species isolated from UTI-suspected patients who attended Bahir Dar City health institutions, Bahir Dar, Northwest Ethiopia, 2019.

*Klebsiella* isolates	Sensitivity	Antimicrobials tested for										
		AMP, *n* (%)	GEN, *n* (%)	AMC, *n* (%)	CXT, *n* (%)	CTX, *n* (%)	CIP, *n* (%)	MER, *n* (%)	COT, *n* (%)	CAZ, *n* (%)	CHL, *n* (%)	NIT, *n* (%)
*K. pneumoniae* (*n* = 25)	S	6 (24)	11 (44)	1 (4)	15 (60)	15 (60)	16 (64)	22 (88)	5 (20)	17 (68)	18 (72)	13 (52)
	I	0	6 (24)	4 (16)	3 (12)	4 (16)	4 (16)	1 (4)	1 (4.)	3 (12)	4 (16)	5 (20)
	R	19 (76)	8 (32)	20 (80)	7 (28)	6 (24)	5 (20)	2 (8)	19 (76)	5 (20)	3 (12)	7 (28)

*K. ozaenae* (*n* = 6)	S	0	5 (83.3)	1 (16.7)	5 (83.3)	4 (66.7)	4 (66.7)	6 (100)	1 (16.7)	5 (83.3)	5 (83.3)	5 (83.3)
	I	0	0	0	00	00	1 (16.7)	0	0	00	0	0
	R	6 (100)	1 (16.7)	5 (83.3)	1 (16.7)	2 (33.3)	1 (16.7)	0	5 (83.3)	1 (16.7)	1 (16.7)	1 (16.7)

*K. oxytoca* (*n* = 4)	S	0	3 (75)	3 (75)	2 (50)	3 (75)	3 (75)	4 (100)	0	3 (75)	3 (75)	3 (75)
	I	0	1 (25)	0	1 (25)	1 (25)	1 (25)	0	2 (50)	0	0	0
	R	4 (100)	00	1 (25)	1 (25)	00	00	0	2 (50)	1 (25)	1 (25)	1 (25)

*K. rhinoscleromatis* (*n* = 3)	S	1 (33.3)	2 (66.7)	1 (33.3)	3 (100)	3 (100)	2 (66.7)	2 (66.7)	1 (33.3)	2 (66.7)	2 (66.7)	2 (66.7)
	I	0	0	0	0	0	0	0	0	0	0	0
	R	2 (66.7)	1 (33.3)	2 (66.7)	0	0	1 (33.3)	1 (33.)	2 (66.7)	33.3	1 (33.3)	1 (33.3)

AMP = ampicillin; GEN = gentamicin; AMC = Augmentin; CXT = cefoxitin; CTX = cefotaxime; CIP = ciprofloxacin; MER = meropenem; COT = cotrimoxazole; CAZ = ceftazidime; CHL = chloramphenicol; NIT = nitrofurantoin; S = sensitive; I = intermediate; R = resistant.

**Table 3:** Multidrug-resistant profile of *Klebsiella* species isolated from UTI-suspected patients who attended Bahir Dar City health institutions, Bahir Dar, Northwest Ethiopia, 2019.

Antimicrobial pattern	Bacterial isolates				Total
	*K. pneumoniae* (*n* = 25)	*K. ozaenae* (*n* = 6)	*K. rhinoscleromatis* (*n* = 3)	*K. oxytoca* (*n* = 4)	
AMP, AMC, COT	5	3	2		10
AMP, AMC, CTX		1		1	2
AMP, AMC, CHL				1	1
AMP, AMC, COT, NIT				1	1
AMP, AMC, COT, CHL	1				1
AMP, AMC, COT, CIP		1			1
AMP, GEN, AMC, COT	3				3
CIP, NIT, GEN, MER, CHL			1		1
AMP, AMC, CTX, CIP, COT	2				2
AMP, AMC, CTX, COT, NIT,	1				1
AMP, GEN, AUG, CTX, CIP, COT, NIT	1				1
AMP, NIT, GEN, AUG, CTX, CIPMER, COT, CAZ, CHL,	2				2
Total	15/25	5/6	3/3	3/4	26/38

**Table 4:** ESBL patterns of *Klebsiella pneumoniae* isolated from UTI-suspected patients who attended Bahir Dar City health institutions, Bahir Dar, Northwest Ethiopia, 2019.

Isolate	Antimicrobials	Interpretation	ESBL production patterns		Total
			Positive	Negative	
*Klebsiella pneumoniae*	Cefotaxime	Sensitive	1	14	15
		Intermediate	3	1	4
		Resistance	6	0	6
		Total	10	15	25
	Ceftazidime	Sensitive	3	14	17
		Intermediate	2	1	3
		Resistance	5	0	5
		Total	10	15	25

**Table 5:** Predictors of urinary tract infections in UTI-suspected patients who attended Bahir Dar City health institutions, Bahir Dar, Northwest Ethiopia, 2019.

Variables		Culture result		COR (95%CI)	*p* value	AOR (95% CI)	*p* value
		Positive, *n* (%)	Negative, *n* (%)				
Sex	Male	16 (8.9)	164 (91.1)	0.8 (0.41–1.59)	0.546.	--	-
	Female	22 (10.7)	183 (91.1)	1	-	-	-

Age (years)	<5	2 (10)	18 (80)	1	-	-	-
	6–15	7 (16)	37 (82)	1.8 (0.29–11.03)	0.525	-	-
	16–30	10 (6.84)	136 (93.15)	1.057 (0.28–4.05)	0.935	-	-
	31–45	7 (7.2)	89 (92.8)	2.720 (779–9.503)	0.117	-	-
	46–60	8 (14.5)	47 (85.5)	2.543	0.166	-	-
	>60	4 (16.7	20 (83.3)	1.175	0.809	-	-

Health facility	FHCSH^*∗*^	25 (9)	253 (91)	1	-	-	-
	GPGH^*∗∗*^	8 (9.2)	79 (9.8)	3.37 (1.13–10.06)	0.029		
	Bahir Dar Health Center	5 (33.3)	15 (72.7)	3.29 (.95–11.45)	0.061		

Residence	Urban	21 (8.97)	213 (91.3)	0.78 (0.39–1.53)	0.464	-	-
	Rural	17 (11.2)	134 (88.8)	1	-	-	-

Educational statues	Illiterate	13 (13.8)	81 (86.2)	1			
	Primary	16 (18.6)	70 (81.4)	0.70 (0.32–1.56)	0.386		0.08
	Secondary	4 (3.9)	103 (96.1)	4.1 (1.93–13.16)	0.016		0.21
	College and above	5 (5.1)	93 (6.9)	2.99 (1.02–8.7)	0.046		0.07

Occupational status	Merchant	3 (10.3)	26 (89.7)	1	-	-	-
	Housewife	13 (11.6)	99 (88.4)	0.80 (0.19–3.4)	0.764	-	-
	Governmental	5 (4.9)	97 (95.1)	0.70 (0.25–1.94)	0.497	-	-
	Farmer	11 (15.5)	60 (84.5)	1.79 (0.53–6.1)	0.352	-	-
	Others	6 (8.5)	65 (91.5)	0.50 (0.18–1.45)	0.202	-	-

Previous antibiotic use	Yes	28 (16.9)	138 (83.1)	4.224 (1.99–9.01)	0.001	2.6 (1.2–6.13)	0.03
	No	10 (4.6)	209 (96.4)	1	-	-	-

Previous hospitalization	Yes	16 (37.2)	27 (62.8)	8.62 (4.05–18.3)	0.001	5.87 (2.4–14.6)	0.001
	No	22 (6.4)	320 (93.6)	1	-	-	-

Patient dept	OPD	29 (8.3)	322 (91.7)	0.250 (0.11–.59)	0.001		
	IPD	9 (26.5)	25 (73.3)	1	-	-	-

Previous UTI	Yes	17 (19.1)	72 (80.9)	3.09 (1.55–6.17)	0.001		
	No	21 (7.1)	275 (92.9)	1	-	-	-

Catheter history	Yes	2 (33.3)	4 (66.7)	4.76 (0.84–26.92)	0.077		
	No	36 (9.5)	343 (90.5)	1	-	-	-

Tight dressing	Yes	4 (66.7)	2 (33.3)	20.29 (3.59–144.9)	0.001	15.0 (1.7–139)	0.02
	No	34 (9)	345 (90)	1	-	-	-

^*∗*^Felege-Hiwot Comprehensive Specialized Hospital; ^*∗∗*^Gammby Private General Hospital.

## Data Availability

All the data supporting the conclusion of the study are included in the paper.
